# Inhibitory Effects of Green Tea Polyphenols on Microbial Metabolism of Aromatic Amino Acids in Humans Revealed by Metabolomic Analysis

**DOI:** 10.3390/metabo9050096

**Published:** 2019-05-11

**Authors:** Yuyin Zhou, Ningning Zhang, Andrea Y. Arikawa, Chi Chen

**Affiliations:** 1 Department of Food Science and Nutrition, University of Minnesota, St. Paul, MN 55108, USA; zhou0882@umn.edu (Y.Z.); znnfst@163.com (N.Z.); 2 Department of Nutrition & Dietetics, University of North Florida, Jacksonville, FL 32224, USA; andrea.arikawa@unf.edu

**Keywords:** green tea polyphenols, metabolome, microbial metabolism, aromatic amino acids

## Abstract

The bioactivities and potential health benefits of green tea polyphenols (GTP) have been extensively investigated, but the metabolic impact of chronic GTP intake on humans is not well defined. In this study, fecal and urine samples from postmenopausal female subjects taking a GTP supplement or placebo for 12 months were compared by liquid chromatography-mass spectrometry-based metabolomic analysis. The GTP-derived and GTP-responsive metabolites were identified and characterized by structural elucidation and quantitative analysis of the metabolites contributing to the separation of control and treatment samples in the multivariate models. Major GTP and their direct sulfate and glucuronide metabolites were absent in feces and urine. In contrast, GTP-derived phenyl-γ-valerlactone and phenylvaleric acid metabolites were identified as the most abundant GTP-derived metabolites in feces and urine, suggesting extensive microbial biotransformation of GTP in humans. Interestingly, GTP decreased the levels of microbial metabolites of aromatic amino acids (AAA), including indoxyl sulfate, phenylacetylglutamine, and hippuric acid, in urine. However, it did not affect the levels of AAA, as well as other microbial metabolites, including short-chain fatty acids and secondary bile acids, in feces. 16S rRNA gene sequencing indicated that the fecal microbiome was not significantly affected by chronic consumption of GTP. Overall, microbial metabolism is responsible for the formation of GTP metabolites while GTP metabolism may inhibit the formation of AAA metabolites from microbial metabolism. Because these GTP-derived and GTP-responsive metabolites have diverse bioactivities, microbial metabolism of GTP and AAA may play important roles in the beneficial health effects of green tea consumption in humans.

## 1. Introduction

Tea (*Camellia sinensis*) is the second most consumed beverage in the world after water [1]. Green tea, as unfermented tea leaf, contains green tea polyphenols (GTP), which are a series of catechins (flavan-3-ols). Epigallocatechin-3-gallate (EGCG) is the major component of GTP while epigallocatechin (EGC), epicatechin-3-gallate (ECG), and epicatechin (EC) are minor components [2]. Like many other polyphenols, GTP are hydrophilic and the substrates of efflux intestinal transporters [3]. These properties limit the bioavailability of GTP by decreasing the absorption in the small intestine and increasing the biliary excretion in the liver. This was confirmed by the fact that only trace amounts of EGCG and ECG were detected in the circulatory system after green tea consumption, and substantial quantities of GTP passed through the small intestine unabsorbed when analyzing ileal fluid collected from ileostomists after the ingestion of GTP [4,5]. Therefore, significant amounts of ingested GTP are subjected to the enzymatic activities of the gut microbial community after reaching the colon [6,7,8]. Human, animal and *in vitro* studies have shown that the colonic microbiota can convert GTP into a series of phenolic metabolites, which were then present in urine and feces after green tea consumption [9,10,11]. These GTP metabolites in humans were detected from short-term trials (24 h after a single treatment) [12,13,14]. The identification of GTP metabolites from long-term treatment in humans has not been reported.

Besides being the substrates of gut microbiota, unabsorbed GTP in the large intestine may have the potential to affect gut microbiota. Short-term GTP treatment and in vitro studies have shown that GTP can inhibit the growth of pathogenic bacteria, such as *Clostridium difficile* and *Staphylococcus *spp., while stimulating the growth of beneficial commensal bacteria, such as *Bifidobacterium *spp. [15,16,17]. However, the effects of long-term GTP on human gut microbiota composition were not fully examined, with only one study showing no significant change of gut microbial composition after 12-week GTP supplementation [18]. Since a relationship between the role of gut microbiota and the beneficial health effects of GTP has been proposed [19], understanding the bi-directional interaction between GTP and gut microbiota is a key step to unraveling the mechanisms underlying the beneficial effects.

The Minnesota green tea trial was a randomized, placebo-controlled, double-blinded trial in which green tea extract was consumed daily for 12 months by healthy postmenopausal women [20]. Findings from this trial showed that GTP supplementation did not affect adiposity or bone mineral density [21], but decreased fasting insulin concentrations in those with elevated baseline levels [22]. In addition, the high-activity genotype of catechol-*O*-methyltransferase (COMT) was positively correlated with the post-prandial serum insulin response in the overweight and obese subjects taking the GTP supplement [23]. All these results suggested that the GTP supplement had influences on the metabolic system. In the current study, samples collected from the Minnesota green tea trial were used to determine the influences of GTP on fecal and urinary metabolomes through liquid chromatography-mass spectrometry (LC-MS)-based metabolomic analysis.

## 2. Results

### 2.1. Influences of GTP on Fecal Metabolome

**Figure 1:**
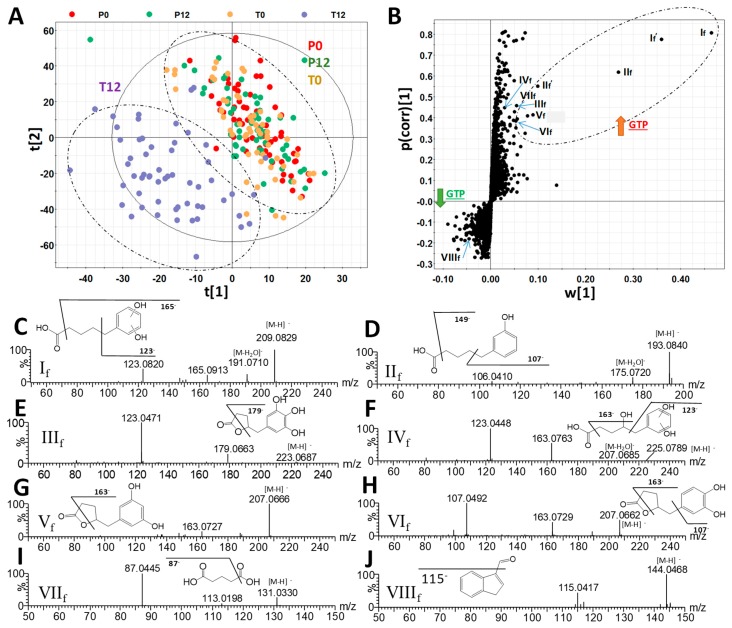
Identification of green tea polyphenol (GTP)-derived and GTP-responsive metabolites in human feces. (**A**) The scores plot from a PLS-DA model on 4 groups of human fecal samples, including P0, P12, T0, and T12. The t[1] and t[2] are the projection values of each sample in the first and second principal components of the model, respectively (*r*^2^ = 0.522 and *q*^2^ = 0.126 for t[1]; *r*^2^ = 0.234 and *q*^2^ = 0.355 for t[2]). (**B**) The S-loadings plot of an OPLS-DA model on the comparison of T12 vs. P0, P12, and T0 fecal samples (*r*^2^ = 0.856 and *q*^2^ = 0.521). Major ions contributing to the separation of T12 from P0, P12, and T0 samples are labeled. (**C**–**J**) Structure and MSMS spectra of I_f_ - VIII_f_ (Table 1).

The chemical compositions of 4 groups of human fecal samples, including P0 (before placebo treatment), P12 (after 12-month placebo treatment), T0 (before GTP treatment), and T12 (after 12-month GTP treatment) were compared by the LC-MS analysis and multivariate analysis. The distribution of fecal samples in the scores plot of a unsupervised principal components analysis (PCA) model did not show clear separation among the 4 treatment groups, indicating that major components of human fecal metabolome were not dramatically altered after one-year GTP treatment (Figure S1). Nevertheless, subtle changes in fecal metabolome were observed based on the separation of T12 group from T0, P0, and P12 groups in the scores plot of a supervised partial least squares-discriminant analysis (PLS-DA) model (Figure 1A). Subsequent analysis of MS signals contributing to this separation in the S-plot from an orthogonal projections to latent structures-discriminant analysis (OPLS-DA) model led to the identification of fecal metabolites that were both increased and decreased by GTP treatment (Figure 1B). A group of hydroxyphenolic compounds, including 5-(dihydroxyphenyl)-valeric acid (I_f_), 3-hydroxyphenyl-valeric acid (II_f_), 5-(3′,4′,5′-trihydroxyphenyl)-γ-valerolactone (III_f_), 4-hydroxy-5-(dihydroxyphenyl)-valeric acid (IV_f_), and 5-(3′,5′-dihydroxyphenyl)-γ-valerolactone (V_f_) and 5-(3′,4′-dihydroxyphenyl)-γ-valerolactone (VI_f_), were identified as the most prominent fecal metabolites increased by GTP treatment (Table 1, Figure S2A). Among these GTP metabolites, the structure of 3-hydroxyphenyl-valeric acid (II_f_) was confirmed by the authentic standard. Others (I_f_, III_f_-VI_f_) were elucidated by accurate mass-based elemental composition analysis, their tandem mass (MSMS) fragmentograms (Figure 1C–H), and their reported presence in the fecal samples from the human and animals treated with green tea extracts [24,25]. Identification of isomers V_f_ and VI_f_ was also based on a previous report on their elution sequence in C18 column [26]. 

**Figure 2:**
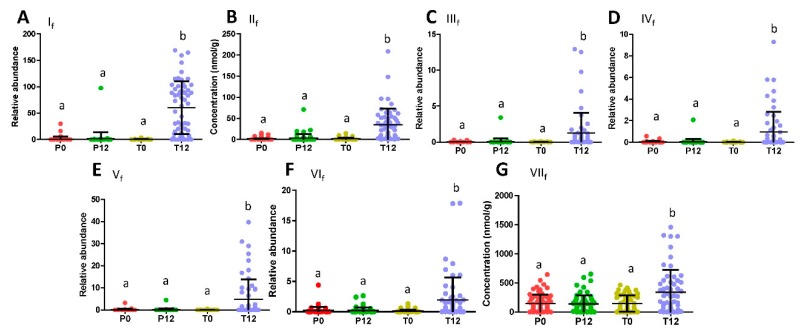
Distribution of GTP-derived and GTP-responsive fecal metabolites in 4 treatment groups. (**A**) Relative abundance of 5-(dihydroxyphenyl)-valeric acid (I_f_). (**B**) Concentration of 3-hydroxyphenyl-valeric acid (II_f_). (**C**) Relative abundance of, 5-(3′,4′,5′-trihydroxyphenyl)-γ-valerolactone (III_f_). (**D**) Relative abundance of 4-hydroxy-5-(dihydroxyphenyl)-valeric acid (IV_f_). (**E**) Relative abundance of 5-(3′,5′-Dihydroxyphenyl)-γ-valerolactone (V_f_). (**F**) Relative abundance of 5-(3′,4′-Dihydroxyphenyl)-γ-valerolactone (VI_f_). (**G**) Concentration of glutaric acid (VII_f_). (Labels a and b indicate statistical difference with a *p* < 0.05 from one-way ANOVA and Tukey’s multiple comparison tests.).

Subsequent quantitative analysis showed that these phenyl-γ-valerolactones and phenylvaleric acids were absent in most P0, P12, T0 samples, but present in most T12 samples (Figure 2A–F). In contrast to the presence of these GTP-derived microbial metabolites, major polyphenols in the GTP preparation, EGCG, EGC, ECG, and EC, were absent in T12 fecal samples (Figure S2), suggesting that extensive microbial degradation occurred to these polyphenols. Besides phenyl metabolites, glutaric acid (VII_f_) was also identified as a fecal metabolite increased by GTP treatment by comparing to the authentic standard (Table 1 and Figure 1I), and then confirmed by quantitative analysis (Figure 2G). Furthermore, indole-3-carboxyaldehyde (VIII_f_) was identified by comparing to the authentic standard among the signals that were negatively correlated to GTP treatment (Table 1 and Figure 1J), but quantitative analysis showed that its level in feces was not significantly affected by the treatment (*p* = 0.2280).

### 2.2. Influences of GTP on Urine Metabolome

**Figure 3:**
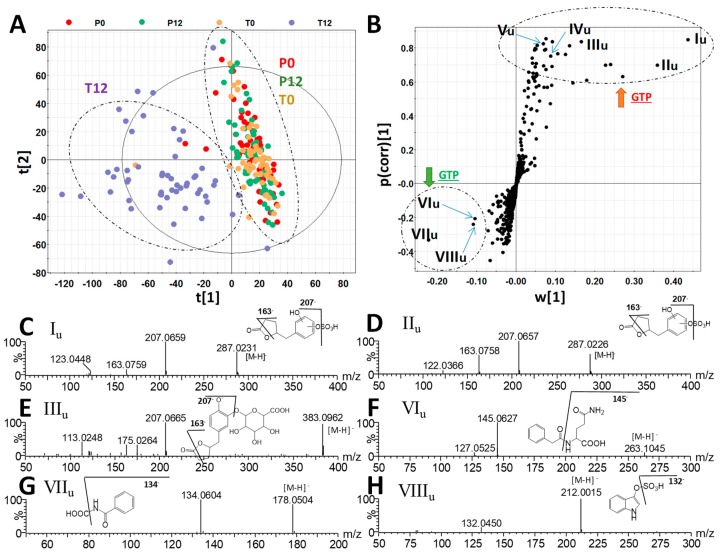
Identification GTP-derived and GTP-responsive metabolites in human urine. (**A**) The scores plot from a PLS-DA model on 4 groups of human urine samples, including P0, P12, T0, and T12. The t[1] and t[2] are the projection values of each sample in the first and second principal components of the model, respectively (*r*^2^ = 0.697 and *q*^2^ = 0.653 for t[1]; *r*^2^ = 0.060 and *q*^2^ = 0.077 for t[2]). (**B**) The S-loadings plot of an OPLS-DA model on the comparison of T12 vs. P0, P12, and T0 urine samples (*r*^2^ = 0.822 and *q*^2^ = 0.721). Major ions contributing to the separation of T12 from P0, P12, and T0 samples are labeled. (**C**–**H**) Structure and MSMS spectra of I_u_–VIII_u_ (Table 2).

To determine whether GTP affected post-absorption metabolome, urine samples from 4 groups were also examined by liquid chromatography-mass spectrometry (LC-MS) metabolomics analysis. Different to the results on fecal metabolome, GTP treatment-based separation of sample groups was visible in both the unsupervised PCA model (Figure S3) and supervised PLS-DA model (Figure 3A). Urinary metabolites contributing to the separation of the T12 group from the other three groups were identified in the S-plot of an OPLS-DA model (Table 2 and Figure 3B). Two 5-(dihydroxyphenyl)-γ-valerolactone sulfate metabolites (I_u_ and II_u_), 5-(dihydroxyphenyl)-γ-valerolactone glucuronide (III_u_), methyl epicatechin sulfate (IV_u_), and methyl epigallocatechin glucuronide (V_u_) were identified as urinary metabolites increased by GTP treatment. Their structures were defined based on accurate mass-based elemental composition analysis, MSMS fragmentograms (Figure 3C–E), and their reported presence in human urine after green tea consumption [25].

**Figure 4:**
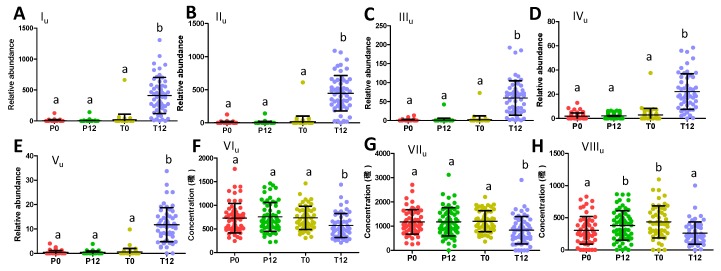
Distribution of GTP-related urine metabolites in 4 treatment groups. (**A**) Relative abundance of 5-(dihydroxyphenyl)-γ-valerolactone sulfate (I_u_). (**B**) Relative abundance of 5-(dihydroxyphenyl)-γ-valerolactone sulfate (II_u_). (**C**) Relative abundance of 5-(dihydroxyphenyl)-γ-valerolactone glucuronide (III_u_). (**D**) Relative abundance of methyl epicatechin sulfate (IV_u_). (**E**) Relative abundance of methyl epigallocatechin glucuronide (V_u_). (**F**) Concentration of phenylacetylglutamine (VI_u_). (**G**) Concentration of hippuric acid (VII_u_). (**H**) Concentration of indoxyl sulfate (VIII_u_). (Labels a and b indicate whether statistical difference (*p* < 0.05) between 2 sample groups from one-way ANOVA and Tukey’s multiple comparison tests.).

Quantitative analysis of their concentrations or relative abundances in urine further showed that three phenyl-γ-valerolactone metabolites (I_u_-III_u_) and two catechin metabolites (IV_u_-V_u_) were absent in most P0, P12, T0 samples, but present in most T12 samples (Figure 4A–E). On the other hand, phenylacetylglutamine (VI_u_), hippuric acid (VII_u_), and indoxyl sulfate (VIII_u_), three well-known bacterial metabolites of aromatic amino acids (AAA) that were identified all by comparing to authentic standards, were found to be negatively correlated with GTP treatment (Figure 4F–H). Quantitative analysis further confirmed that GTP significantly decreased the level of these microbial metabolites in T12 feces (*p* = 0.003 for VI_u_; *p* = 0.0005 for VII_u_; *p* = 0.0002 for VIII_u_) (Figure 4F–H). 

### 2.3. Influences of GTP on Gut Microbiome

**Figure 5:**
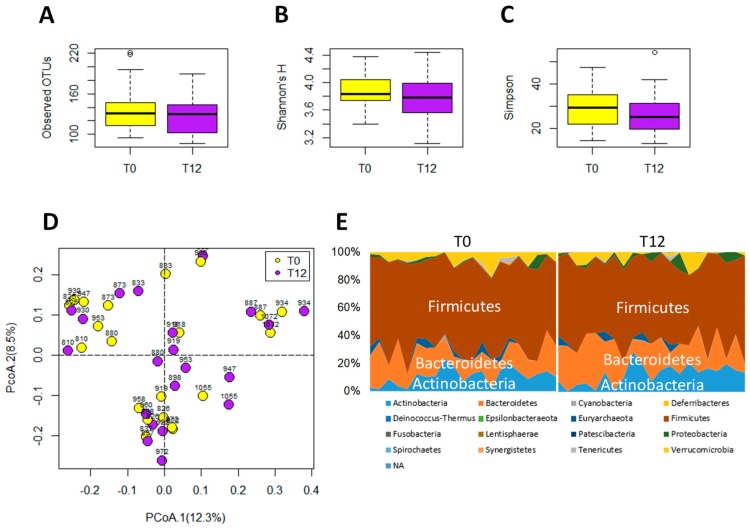
Microbiome analysis of paired T0 and T12 fecal samples. (**A**) Number of operational taxonomic units (OTU) detected by 16S rRNA gene analysis. (**B**) Shannon’s H index. (**C**) Simpson index. (**D**) Distribution of paired T0 and T12 samples with their ID numbers in a principal coordinates analysis (PCoA) model on fecal microbiome. (**E**) Relative abundances of individual phyla in T0 and T12 samples.

**Figure 6:**
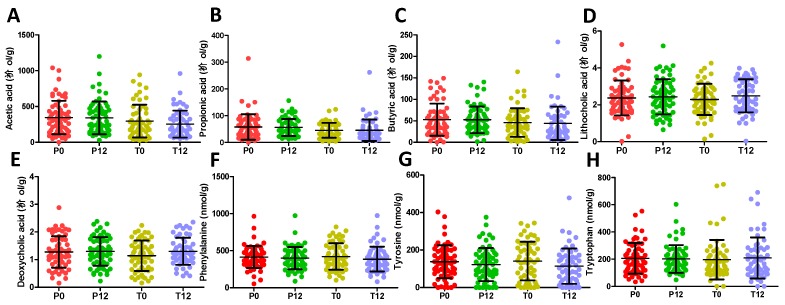
Concentrations of short chain fatty acids, secondary bile acids, and AAA in P0, P12, T0, and T12 fecal samples. (**A**) Acetic acid. (**B**) Propionic acid. (**C**) Butyric acid. (**D**) Lithocholic acid. (**E**) Deoxycholic acid. (**F**) Phenylalanine. (**G**) Tyrosine. (**H**) Tryptophan.

The 16S rRNA gene analysis of feces samples from 21 paired T0 and T12 subjects was conducted to determine whether GTP treatment affected the gut microbiota of human subjects. The results showed that GTP did not affect the fecal microbial richness since the numbers of detected operational taxonomic units (OTUs), which were based on the average of 21,805 reads per sample (ranging from 5426 to 35,160), did not differ between T0 and T12 samples (*p* = 0.435, Figure 5A). Both Shannon H indexes and Simpson indexes of T0 and T12 groups showed that GTP treatment did not alter the α diversity of fecal microbiome (*p* = 0.294 for Shannon H index; *p* = 0.33 for Simpson index, Figure 5B–C). Principal coordinates analysis (PCoA) on a Bray-Curtis dissimilarity matrix on gut microbial communities also could not separate T0 and T12 groups in the model (Figure 5D). Interestingly, many paired T0 and T12 samples from the same subjects remained close to each other in the model (Figure 5D), suggesting the stability of their fecal microbial profile during one-year GTP treatment. Permutational multivariate analysis of variance (PERMANOVA), which is the analysis of variance using distance matrices and permutation tests, also showed no significant difference between T0 and T12 groups (*p* = 0.992). The relative abundances of Firmicutes, Bacteroidetes, and Actinobacteria, the dominating phyla in fecal microbiome (Figure 5E), did not differ between two groups, (Wilcoxon rank-sum test, the false discovery rate (FDR)-corrected *Q*-value = 0.64 for Firmicutes, 0.64 for Bacteroidetes, and 0.65 for Actinobacteria, respectively). In addition, no significant differences were observed on class, family, and genus levels (data not shown). The status of fecal microbiome was further examined by measuring the levels of major microbial metabolites, including short chain fatty acids (SCFAs) and secondary bile acids (deoxycholic acid and lithocholic acid), in feces. The results indicated that GTP treatment did not significantly affect their concentrations (Figure 6A–E). In addition, to investigate whether or not the decreased microbial metabolites of AAA were due to the reduced availability of dietary AAA, the levels of three AAA in feces were determined. No significant difference was detected among all 4 treatment groups (Figure 6F–H).

## 3. Discussion

Based on their structures and origins, the fecal and urinary metabolites affected by 1-year GTP treatment can be grouped as GTP-derived metabolites and GTP-responsive metabolites. The routes of their formation and their potential roles in the bioactivities of GTP were discussed as follows.

### 3.1. Formation and Potential Functions of GTP-Derived Metabolites in Human Feces and Urine 

**Figure 7:**
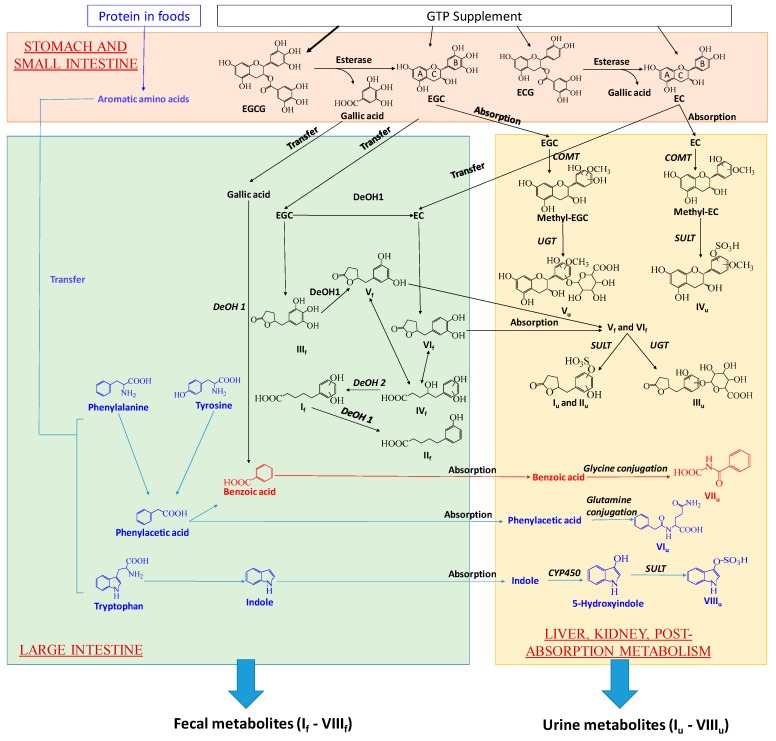
Proposed major metabolic pathways for forming GTP-derived metabolites and GTP-responsive metabolites in feces and urine by gut microbiota and human metabolism system. Metabolic pathways shared by GTP and AAA are in red. DeOH: dehydroxylation.

A prominent observation of this study was the absence of unmetabolized GTP in examined human urine and fecal samples, suggesting that EGCG, EGC, ECG, and EC, no matter absorbed or unabsorbed, were thoroughly metabolized after the intake. Subsequent analyses of GTP metabolites on their structures and their distribution in human feces and urine implicated the joint contributions of digestion, microbial metabolism and post-absorption metabolism to their formation in human body. The proposed biotransformation pathways for producing fecal and urinary GTP metabolites were based on these results as well as the existing knowledge on how human body and gut microbiota dispose xenobiotics (Figure 7). 

Formation of GTP-derived fecal metabolites: Hydrolysis, ring opening and fission, and dehydroxylation are the major biotransformation reactions responsible for yielding a series of phenyl-γ-valerlactone and phenylvaleric acid metabolites of GTP in feces. In the alimentary canal, unabsorbed EGCG and ECG were hydrolyzed by the esterases in saliva, gastric and pancreatic juices, and microbiota to form EGC and EC, respectively, and release gallic acid [10,11,27,28]. EC can also be formed by the microbial dehydroxylation of EGC [10]. Microbial metabolism of the flavan-3-ol skeleton of EGC, including reductive opening of C ring and subsequent fission of the phloroglucinol moiety of A ring, produced 5-(3′,4′,5′-trihydroxyphenyl)-γ-valerolactone (III_f_) (Figure 7) [10,12,29]. III_f_ could undergo further microbial dehydroxylation at 4′ position on the phenolic ring, forming 5-(3′,5′-trihydroxyphenyl)-γ-valerolactone (V_f_) [10,29,30]. EC could undergo similar microbial reactions of C ring opening and A ring fission, producing 5-(3′,4′-dihydroxyphenyl)-γ-valerolactone (VI_f_) (Figure 7) [12]. The γ-valerolactone ring of V_f_ and VI_f_ could be opened through hydrolysis, yielding 4-hydroxy-5-(dihydroxyphenyl)-valeric acid (IV_f_) [10,12,30]. Microbial dehydroxylation reactions could further convert IV_f_ to 5-(dihydroxyphenyl)-valeric acid (I_f_) and then to 3-hydroxyphenyl-valeric acid (II_f_) [10]. Among these 6 fecal GTP metabolites (I_f_–VI_f_), I_f_ has been reported as the major catabolite in rat feces after green tea intake [10], and detected in human feces after red wine consumption [31]. III_f_, V_f_ and VI_f_ were previously reported to be produced by humans, rats and mice [9,10,30]. However, II_f_ as a major bacterial catabolite of GTP in human feces has not been reported previously.

Formation of GTP-derived urinary metabolites: Absorbed EGC, either directly from the GTP supplement, or from the hydrolysis of EGCG, likely underwent COMT-mediated methylation followed by uridine 5’-diphospho-glucuronosyltransferase (UGT)-mediated glucuronidation to form methyl epigallocatechin glucuronide (V_u_). Similarly, absorbed EC was metabolized by COMT-mediated methylation and sulfotransferase (SULT)-mediated sulfation reactions to form methyl epicatechin sulfate (IV_u_). The presence of methylated EC and EGC metabolites (IV_u_ and V_u_), but not the methylated ECG and EGCG metabolites, in urine was consistent with the reported bioavailability of EC (31%) and EGC (14%) in rats, which were significantly greater than that of EGCG (< 1%) [32]. Moreover, the detection of two 5-(dihydroxyphenyl)-γ-valerolactone glucuronide (I_u_ and II_u_) and 5-(dihydroxyphenyl)-γ-valerolactone glucuronide (III_u_) indicated the absorption of V_f_ and VI_f_ in the large intestine. These hydoxylphenyl-γ-valerolactone conjugates, together with conjugated and methylated EC and EGC, had been previously reported as GTP metabolites in human urine after green tea intake [12].

Potential roles of GTP-derived metabolites in reported bioactivities of GTP: Potential beneficial health effects of GTP, including an anti-inflammatory effect, cardioprotection, cancer prevention, neuroprotection, and body weight control, have been documented by numerous in vitro and animal studies [33,34], but the chemical entities responsible for these effects were not well defined. The results of this study clearly showed that microbial metabolites of GTP, mainly phenyl-γ-valerolactones and phenylvaleric acids, are far more abundant than non-microbial metabolites of GTP based on their relative abundances in human urine (Figure 4A–E). Therefore, the microbial metabolites of GTP are more likely responsible for the beneficial effects of GTP. This conclusion is supported by the reported bioactivities of phenyl-γ-valerolactones and phenylvaleric acids in recent studies. Anti-inflammatory activities of phenyl-γ-valerolactones were demonstrated through *in vitro* studies, in which both trihydroxyphenyl-γ-valerolactone (III_f_) and 5-(3′,4′-dihydroxyphenyl)-γ-valerolactones (VI_f_) decreased nitric oxide release in murine macrophages [35,36], and 5-(3′,4′-dihydroxyphenyl)-γ-valerolactones (VI_f_) also decreased tumor necrosis factor α (TNFα)- induced nuclear factor kappa-light-chain-enhancer of activated B cells (NF-kB) transcriptional activity in transfected HepG2 cells [37]. Potential cardioprotective effects of dihydroxyphenyl-γ-valerolactones (V_f_ and VI_f_) have also been observed since 5-(3′,5′-dihydroxyphenyl)-γ-valerolactone (V_f_) significantly decreased systolic blood pressure in a hypertensive rat model [38], and the plasma level of 5-(dihydroxyphenyl)-γ-valerolactone-4′-sulfate was well correlated with flow-mediated vasodilation on healthy human subjects taking cranberry drinks [39]. Moreover, the cancer prevention effect of 5-(3′,4′-dihydroxyphenyl) valeric acid (one possible structure of I_f_), 4-hydroxy-5-(3′,4′,5′-trihydroxyphenyl) valeric acid, and 5-(3′,4′,5′-trihydroxyphenyl) valeric acid was observed through their inhibitory activity on cancer cells [40]. Despite these promising results, the information on the bioactivites of these phenyl-γ-valerolactones and phenylvaleric acids remains limited. Further studies are needed to understand the disposition (absorption, distribution, metabolism, and excretion) of these microbial metabolites of GTP inside the body, and their roles in the beneficial health effects of GTP.

### 3.2. Potential Causes and Consequences of GTP-Elicited Decreases in Microbial Metabolism of AAA

Following the detection of microbial metabolites of GTP in feces and urine, the influences of 1-year GTP treatment on gut microbiota were examined through both metabolomic and microbiomic analyses in this study. Microbiomic analysis showed that chronic GTP treatment did not influence the overall gut microbiome profile. This result is consistent with the conclusion of a recent human study, in which the subjects consumed no less than 540 mg of EGCG per day for 12 weeks [18]. However, another two short-term GTP human trials have shown that GTP inhibited the growth of *Clostridium spp.* [16], while it stimulated the growth of bifidobacteria [17]. The dose of GTP in one study (0.4 g/subject, 3 times per day) was comparable to the dose of our study, but the trial only lasted for four weeks, which was much shorter than our study, and the microfloral analysis was conducted with targeted cultures of bacterial colonies, instead of sequencing [16]. In the other short-term study, the subjects consumed 1 liter of green tea drink per day for 10 days, and microbial analysis only examined *Bifidobacterium* species [17]. Considering the limited coverage of microbial analysis in these two studies, it is reasonable to suggest that short-term GTP treatment might selectively affect a small group of microbial species while long-term GTP treatment may have limited influence on the human gut microbiome, potentially through acclimation or another mechanism. This conclusion is partially supported by the metabolomic analysis in our study since SCFAs and secondary bile acids, the most commonly examined functional metabolites from microbial metabolism in the large intestine, were not affected by GTP. Instead, bacterial metabolites of AAA, including phenylacetylglutamine (VI_u_), indoxyl sulfate (VIII_u_), and hippuric acid (VII_u_), were identified as the urinary metabolites that were significantly decreased by GTP, suggesting the existence of selective interactions between AAA metabolism and GTP treatment.

Potential interactions between AAA metabolism and GTP metabolism: The biosynthesis pathways for forming bacterial metabolites of AAA were well studied. Phenylacetylglutamine (VI_u_) is formed by the conjugation of glutamine to phenylacetic acid, a bacterial metabolites of tyrosine and phenylalanine [41], while indoxyl sulfate (VIII_u_) is formed from the oxidation and sulfation of indole, a bacterial metabolite of tryptophan [42]. Benzoic acid, the precursor of hippuric acid (VII_u_), originates from multiple sources, including tyrosine and phenylalanine as well as phenolic compounds in plants [43]. Since digestion, microbial metabolism and post-absorption metabolism are the shared processes in forming microbial metabolites of GTP and AAA in the human body (Figure 7), whether the interactions between AAA metabolism and GTP metabolism occur in these processes is discussed as follows. (1). Interactions might not occur in the digestion since the levels of free AAA in fecal samples did not differ among 4 treatment groups (Figure 6F–H). This observation suggested that the decreases of VI_u_–VIII_u_ were not due to the decreases of their AAA precursors released from protein digestion in the intestine. (2). Interactions might occur in the post-absorption metabolism because of partial overlapping in the sulfation to form GTP metabolites (I_u_, II_u_, IV_u_) and the sulfation to form indoxyl sulfate (VIII_u_). This type of competition on the enzymes and the cofactor of sulfation reactions had been observed between xenobiotics and bacterial amino acid metabolites [44]. However, it might not explain the decreases of phenylacetylglutamine and hippuric acid, which were formed by amino acid conjugations. (3). Interactions likely occurred in the microbial metabolism through both competitive and non-competitive inhibition of bacterial enzymes. Multiple types of reactions, including transamination, oxidative decarboxylation, dehydroxylation, were responsible for producing the intermediates (such as phenylpyruvic acid, 4-OH-phenylpyruvic acid, and indolepyruvic acid) and end products (such as phenylacetic acid, indole-3-carboxaldehyde, indole, and benzoic acid) in the bacterial metabolism of phenylalanine, tyrosine, and tryptophan (Figure 7) [41,45,46,47,48,49]. In the microbial metabolism of GTP, dehydroxylation occurs in the B ring while oxidation and decarboxylation might occur in the fission of the A ring (Figure 7) [50]. Therefore, competitive inhibition could occur if the same enzymes and reactions were shared between microbial metabolism of GTP and AAA. Moreover, it has been shown that GTP, especially EGCG, can function as the inhibitors of diverse dehydrogenases and decarboxylases [51,52,53]. It is possible that GTP-elicited noncompetitive inhibition may suppress similar reactions in bacterial AAA metabolism.

While urinary hippuric acid was found to be significantly decreased by GTP treatment in the current study, other studies have reported the increase of urinary hippuric acid as a metabolic event after GTP consumption [13,14,54]. Differences in GTP treatment between this long-term feeding study (1.3 g GTP/day for 12 months) and previous short-term studies (such as 6 g GTP in 24 h) may contribute to this discrepancy on GTP-elicited changes in urinary hippuric acid since gallic acid from GTP is a direct precursor of hippuric acid. Besides the increases of phenylacetylglutamine (VI_u_), indoxyl sulfate (VIII_u_), and hippuric acid (VII_u_) in urine, the increase of glutaric acid (VII_f_) in feces, a known bacterial metabolite, was observed. Interestingly, the increase of glutaric acid in feces was also observed in humans consuming red wine rich in polyphenols [31]. Since tryptophan and lysine have been identified as the precursors of glutaric acid in bacterial metabolism [55,56], whether the increase of glutaric acid in the current study was associated with altered microbial metabolism of amino acids or other specific metabolic pathways requires further studies.

Potential consequences of inhibiting microbial AAA metabolism: Both indoxyl sulfate (VIII_u_) and phenylacetylglutamine (VI_u_) are known for their roles as uremic toxins in chronic kidney disease (CKD) and the subsequent development of cardiovascular diseases [57]. The cardiotoxicity of indoxyl sulfate and other tryptophan metabolites might be mediated by the activation of an aryl hydrocarbon receptor, which can contribute to the prooxidant, proinflammatory, procoagulant, and pro-apoptotic events in CKD-associated cardiovascular complications [58]. Interestingly, GTP, especially EGCG, have been shown to be protective against CKD [59] and cardiometabolic diseases [60], and these beneficial effects have been largely attributed to the anti-inflammatory and antioxidant activities of GTP. The results from this study suggest that decreasing the levels of uremic toxins might be an additional mechanism behind these protective effects of GTP. Further study examining the effects of GTP on uremic toxin burden in CKD models may provide more insights on the health effects associated with GTP-elicited inhibition of microbial AAA metabolism.

In conclusion, metabolomic analysis of human feces and urine samples in this study revealed extensive contributions of microbial metabolism to the formation of GTP metabolites and the influences of GTP on microbial production of AAA metabolites. Because these GTP-derived and GTP-responsive metabolites had diverse bioactivities, microbial metabolism of GTP and AAA may play important roles in the beneficial health effects of green tea consumption in humans.

## 4. Materials and Methods

### 4.1. Chemicals and Reagents 

LC-MS-grade water, acetonitrile (ACN) and formic acid were purchased from Fisher Scientific (Houston, TX, USA). The metabolite standards used for structural confirmation were from Enamine Chemicals (Kiev, Ukraine) and Sigma-Aldrich (St. Louis, MO, USA), respectively.

### 4.2. Green Tea Polyphenols (GTP) Preparation 

The supplement used in this study is Green Tea Extract Catechin Complex (Corban complex GTB; Investigational New Drug #103,431). It is a decaffeinated green tea extract with roughly 330 ± 29.0 mg total catechins per capsule, including 211 ± 11.0 mg EGCG, 27 ± 30.0 mg EGC, 51 ±19.0 mg ECG, and 27 ± 6.0 mg EC. Placebo capsules were identical in appearance to the green tea extract capsules and contained maltodextrin (50%), cellulose (49.5%), and magnesium stearate (0.5%). Both green tea extract capsules and placebo were supplied by Corban Laboratories/Eniva Nutraceutics (Plymouth, MN) [20].

### 4.3. Study Design 

Subjects were recruited in the Minneapolis-St. Paul metropolitan area, from August 2009 through April 2013. Subjects were healthy postmenopausal females, 50–60 years and diagnosed with heterogeneously (51–75% glandular) or extremely (75% glandular) dense breasts. This population was selected based on their risk for breast cancer and also their potential to receive benefits from GTP intake. All subjects reported no smoking habits, consuming no more than one cup of green tea per week, and less than 7 drinks of alcohol per week. Body mass index (BMI) of all subjects was between 19.3 and 36 kg/m^2^, and their weight was stable (weight change < 10 lbs during the one-year experimental period) [20]. The study was conducted in a randomized, double-blind, placebo-controlled design. Participants were instructed to take two capsules, twice daily with breakfast and dinner, for a daily total of 1315 ± 115.0 mg total catechins containing 843.0 ± 44.0 mg EGCG, or two placebo capsules twice daily for 12 months. The BMI and dietary intake of the participants in placebo and GTP groups were comparable (Tables S1 and S2).

### 4.4. Fecal and Urine Samples Collection 

Participants collected fecal samples and 24 h urine at months 0 and 12. Fecal and urine samples were labeled P0 (from 61 participants before placebo treatment), P12 (from the same 61 participants after 12-month placebo treatment), T0 (from 63 participants before GTP treatment), and T12 (from the same 63 participants after 12-month GTP treatment). Fecal samples were collected by subjects at home, delivered to the clinic within 24 h of collection, and subsequently stored at −80 °C until further processing. Subjects collected all urine for 24 h in a 3 liter plastic container containing 3 g of ascorbic acid. Urine was kept refrigerated until it was brought to the clinic the following day. Urine volume was recorded and aliquots without additives were stored at −80 °C.

### 4.5. Preparation of Fecal and Urine Samples 

Fecal samples were prepared by mixing 50 mg of feces with 500 μL of 50% aqueous ACN containing 5 μM sulfadimethoxine, and then sonicating for 10 min. Sulfadimethoxine was used as the internal standard to monitor the performance of sample preparation and LC-MS analysis. After vortexing, the samples were centrifuged at 18,000 *g* at 4 °C for 10 min to obtain fecal extract supernatants. Urine samples were prepared by mixing 100 μL of urine with 100 μL of ACN containing 0.1% formic acid and 5 μM sulfadimethoxine, and then centrifuging at 18,000 *g* for 10 min to obtain the supernatants. For short-chain fatty acids analysis, 5 μL of the supernatant spiked with 200 µM ^2^D_4_-acetic acid as internal standard was subjected to 2-hydrazinoquinoline (HQ) derivatization by mixing biological sample with a 100 µL of ACN solution containing 1 mM 2,2′-dipyridyl disulfide (DPDS), 1 mM triphenylphosphine (TPP) and 1 mM HQ [61]. The reaction mixture was incubated at 60 °C for 60 min. The HQ derivatives in the reaction mixture were analyzed by LC-MS.

### 4.6. LC−MS Analysis 

A 5 μL sample aliquot was injected into an Acquity Ultraperformance Liquid Chromatography (UPLC) system (Waters, Milford, MA, USA) and separated in a BEH C18 column (Waters, Milford, MA, USA). For fecal sample analysis, 0.05% (*v*/*v*) aqueous acetic acid containing 5 mM ammonium acetate (A), 95% (*v*/*v*) aqueous ACN containing 0.05% acetic acid and 5 mM ammonium acetate (B) were used as mobile phase. For urine sample analysis, 0.1% formic acid (A) and ACN containing 0.1% formic acid (B) were used as mobile phase. The LC eluant was introduced into a Xevo-G2-S quadrupole time-of-flight mass spectrometer (QTOFMS, Waters, Milford, MA, USA) for accurate mass measurement and ion counting. Capillary voltage and cone voltage for electrospray ionization were maintained at 0.1 kV and 5 V respectively for negative-mode detection. Source temperature and desolvation temperature were set at 120 and 350 °C, respectively. Nitrogen was used as both cone gas (50 L/h) and desolvation gas (800 L/h), and argon was used as collision gas. For accurate mass measurement, the mass spectrometer was calibrated with sodium formate solution with a mass-to-charge ratio (*m/z*) of 50−1000 and monitored by intermittent injection of the lock mass leucine enkephalin ([M − H]^−^ = *m/z* 554.2615) in real time. Mass chromatograms and mass spectral data were acquired and processed by MassLynx software (Waters, Milford, MA, USA) in centroided format. The chemical identities of compounds of interest were determined by accurate mass measurement, elemental composition analysis, MSMS fragmentation, and comparisons with authentic standards (if available). Individual compound concentrations were determined by calculating the ratio between the peak area of compound and the peak area of internal standard and fitting with a standard curve using QuanLynx software (Waters, Milford, MA, USA).

### 4.7. Multivariate Analysis and Data Visualization 

After data acquisition in the UPLC−QTOFMS system, the chromatographic and spectral data of samples were deconvoluted by MarkerLynx software (Waters, Milford, MA, USA). A multivariate data matrix containing information on sample identity, ion identity (retention time and *m/z*), and ion abundance was generated through centroiding, deisotoping, filtering, peak recognition, and integration. The intensity of each ion was calculated by normalizing the single-ion counts (SIC) versus the total-ion counts (TIC) in the whole chromatogram. The data matrix was exported into SIMCA-P+ software (Umetrics, Kinnelon, NJ, USA) and transformed by *Pareto* scaling, and then characterized by multivariate data analysis. Both unsupervised principal components analysis (PCA) models and supervised models from partial least squares-discriminant analysis (PLS-DA) and supervised orthogonal partial least squares-discriminant analysis (OPLS-DA) were constructed to delineate the relationship among sample groups, as well as the contribution of each MS signal to the principal components (PCs) of the multivariate model. The compounds contributing to the sample separation in the model were identified based on their p[1] and p(corr)[1] values in the S-plot of OPLS-DA model, which represented their weight and reliability on representing the difference among samples, respectively.

### 4.8. Marker Characterization 

The chemical identities of GTP-derived and GTP-responsive fecal and urine marker metabolites were determined by accurate mass measurement; elemental composition analysis; isotope fitness (i-FIT value); searching the Human Metabolome Database (HMDB), Kyoto Encyclopedia of Genes and Genomes (KEGG) and Lipid Maps databases using MassTRIX search engine (http://masstrix3.helmholtz-muenchen.de/masstrix3/); MSMS fragmentation; and comparisons with authentic standards if available. Authentic standards were used to identify and quantify 3-hydroxyphenyl-valeric acid (II_f_), glutaric acid (VII_f_), indole-3-carboxaldehyde (VIII_f_), phenylacetylglutamine (VI_u_), hipputic acid (VII_u_), and indoxyl sulfate (VIII_u_). The identities of I_f_ and IV_f_ were tentatively determined through accurate mass-based interpretation of MSMS fragmentation and previous publication on their presence as GTP metabolites and their MSMS spectra [24]. A similar approach was used in the characterization of III_f_, V_f_, VI_f_, I_u_-V_u_ as GTP metabolites [25].

### 4.9. Fecal Sample Processing and 16S rRNA Gene Analysis 

Fecal DNA from T0 and T12 samples was extracted individually and purified with Quick-DNA fecal/soil microbe microprep kit (Zymo Research, Orange, CA, USA). DNA quantity was measured using a NanoDrop 2000c spectrophotometer (Thermo Fisher Scientific, Waltham, MA, USA). Only samples with > 5 ng/µL DNA (21 pairs of T0 and 21 T12 fecal samples) were selected for 16S rRNA gene analysis using 454-pyrosequencing. Previously designed PCR primers flanking the V4 hypervariable region of bacterial 16S rRNA gene were used [62]. 16S rRNA gene amplification and sequencing was performed at the University of Minnesota Genomics Center (UMGC) as previously described [63]. The trimmed 16S rRNA gene sequence pairs were quality filtered (*q*-score > 20, using QIIME 1.8.0). Bacterial sequence reads were compared to a reference database of known 16S rRNA genes using the Ribosomal Database Project (RDP) databases (http://rdp.cme.msu.edu/seqmatch). Phylogenetic assignments were performed RDP classifiers. All multivariate and community analysis were conducted using the vegan, labdsv, and ape package in the statistical software R.

### 4.10. Statistical Analysis 

The statistical significance among samples in different groups were analyzed by one-way ANOVA and Tukey’s multiple comparison post hoc test using GraphPad Prism version 5.01 (GraphPad Software, Inc. La Jolla, CA, USA). A *p* value of < 0.05 was considered as statistically significant.

## Figures and Tables

**Table 1:** Markers of GTP treatment in fecal metabolome. Δppm: deviation of the measured mass from the theoretical mass in parts per million.

Ions	Retention Time (min)	*m/z* of [M–H]^-^	Δppm	Formula	Identity
I_f_	3.40	209.0819	2	C_11_H_14_O_4_	5-(Dihydroxyphenyl)-valeric acid
I_f_^’^	3.40	419.1704	0	C_22_H_28_O_8_	5-(Dihydroxyphenyl)-valeric acid in-source dimer
II_f_	4.79	193.0867	1	C_11_H_14_O_3_	3-Hydroxyphenyl-valeric acid
II_f_^’^	4.79	387.1799	2	C_22_H_28_O_6_	3-Hydroxyphenyl-valeric acid in-source dimer
III_f_	2.09	223.0609	1	C_11_H_12_O_5_	5-(3′,4′,5′-Trihydroxyphenyl)-γ-valerolactone
IV_f_	2.01	225.0763	2	C_11_H_14_O_5_	4-Hydroxy-5-(dihydroxyphenyl)-valeric acid
V_f_	2.72	207.0661	1	C_11_H_12_O_4_	5-(3′,5′-Dihydroxyphenyl)-γ-valerolactone
VI_f_	3.05	207.0664	3	C_11_H_12_O_4_	5-(3′,4′-Dihydroxyphenyl)-γ-valerolactone
VII_f_	0.68	131.0341	6	C_5_H_8_O_4_	Glutaric acid
VIII_f_	3.81	144.0449	4	C_9_H_7_NO	Indole-3-carboxaldehyde

**Table 2:** Markers in urine metabolome. Δppm: deviation of the measured mass from the theoretical mass in parts per million.

Ions	RT (min)	*m/z* of [M – H]^-^	Δppm	Formula	Identity
I_u_	2.41	287.0225	2	C_11_H_12_O_7_S	5-(Dihydroxyphenyl)-γ-valerolactone sulfate
II_u_	2.93	287.0225	2	C_11_H_12_O_7_S	5-(Dihydroxyphenyl)-γ-valerolactone sulfate
III_u_	2.10	383.0971	3	C_17_H_20_O_10_	5-(Dihydroxyphenyl)-γ-valerolactone glucuronide
IV_u_	3.29	383.0420	4	C_16_H_16_O_9_S	Methyl epicatechin sulfate
V_u_	2.42	495.1121	4	C_22_H_24_O_13_	Methyl epigallocatechin glucuronide
VI_u_	2.59	263.1031	2	C_13_H_16_N_2_O_4_	Phenylacetylglutamine
VII_u_	2.39	178.0503	3	C_9_H_8_NO_3_	Hippuric acid
VIII_u_	2.42	212.0017	2	C_8_H_7_NO_4_S	Indoxyl sulfate

## Supplementary Materials

The following are available online at https://www.mdpi.com/2218-1989/9/5/96/s1, Table S1: BMI of human subjects in 4 sample groups, Table S2: Dietary intake of human subjects in 4 sample groups, Figure S1: The scores plot from a PCA model on fecal samples, Figure S2: Representative chromatogram of GTP-derived bacterial metabolites in human feces (A) and urine (B), Figure S3: The scores plot from a PCA model on urine samples.
