# Supplementary material for: Inhibitory Effects of Green Tea Polyphenols on Microbial Metabolism of Aromatic Amino Acids in Humans Revealed by Metabolomic Analysis
Source: Metabolites. 2019 May 11;9(5):96. doi: 10.3390/metabo9050096 (PMC6571926; doi:10.3390/metabo9050096)

metabolites

Inhibitory effects of green tea polyphenols on microbial metabolism of aromatic amino acids in humans revealed by metabolomic analysis

Yuyin Zhou[1], Ningning Zhang[1], Andrea Y. Arikawa[2], and Chi Chen[1,*]

[1] Department of Food Science and Nutrition, University of Minnesota, St. Paul, MN 55108; E-Mail: zhou0882@umn.edu (Y.Z.); znnfst@163.com (N.Z.); chichen@umn.edu (C.C.)

[2] Department of Nutrition & Dietetics, University of North Florida, Jacksonville, FL 32224; E-Mail: andrea.arikawa@unf.edu

* Correspondence: chichen@umn.edu. Tel.: +1-612-624-7704; Fax: +1-612-625-5272.

SUPPLEMENTARY DATA

Table S1. BMI of human subjects in 4 sample groups

Treatment	Time	Mean	Std. Error	95% Confidence Interval Lower Bound	Upper Bound
Control	P0	25.549	0.438	24.685	26.413
	P12	25.585	0.435	24.728	26.442
green tea	T0	25.666	0.450	24.780	26.552
	T12	25.667	0.454	24.774	26.561

Table S2. Dietary intake of human subjects in 4 sample groups.[a]

Dietary Variables	Control		Green Tea		*P*-value[b]
	P0	P12	T0	T12	
Calories (kcals)	1545 ± 74	1413 ± 74	1532 ± 76	1434 ± 77	0.963
Carbohydrates (g)	193.1 ± 9.3	174.5 ± 9.3	188.7 ± 9.5	171.2 ± 9.6	0.682
Protein (g)	60.4 ± 3.2	57.1 ± 3.2	62.6 ± 3.3	58.4 ± 3.3	0.592
Fat (g)	58.5 ± 3.6	52.7 ± 3.6	57.4 ± 3.7	57.1 ± 3.8	0.656
Saturated Fat (g)	18.2 ± 1.2	16.4 ± 1.2	18.2 ± 1.2	17.9 ± 1.2	0.548
Cholesterol (mg)	159 ± 10	152 ± 10	159 ± 11	154 ± 11	0.930
Alcohol (g)	6.7 ± 1.0	7.1 ± 1.0	7.6 ± 1.1	6.2 ± 1.1	0.975
Fiber (g)	16.6 ± 1.0	15.3 ± 1.0	17.0 ± 1.0	16.5 ± 1.0	0.455
Glycemic Load	91.4 ± 4.5	82.0 ± 4.5	87.9 ± 4.6	79.4 ± 4.6	0.509
Added sugar (g)	46.6 ± 3.2	38.6 ± 3.2	41.4 ± 3.3	36.0 ± 3.3	0.229

[a] Values are mean ± standard error.

[b] *P*-values are for the comparison between placebo and GTP groups.

Figure S1. The scores plot from a PCA model on 4 groups of human fecal samples, including P0 (before placebo treatment), P12 (after 12-month placebo treatment), T0 (before GTP treatment), and T12 (after 12-month GTP treatment). The t[1] and t[2] are the projection values of each sample in the first and second principal components of the model, respectively.

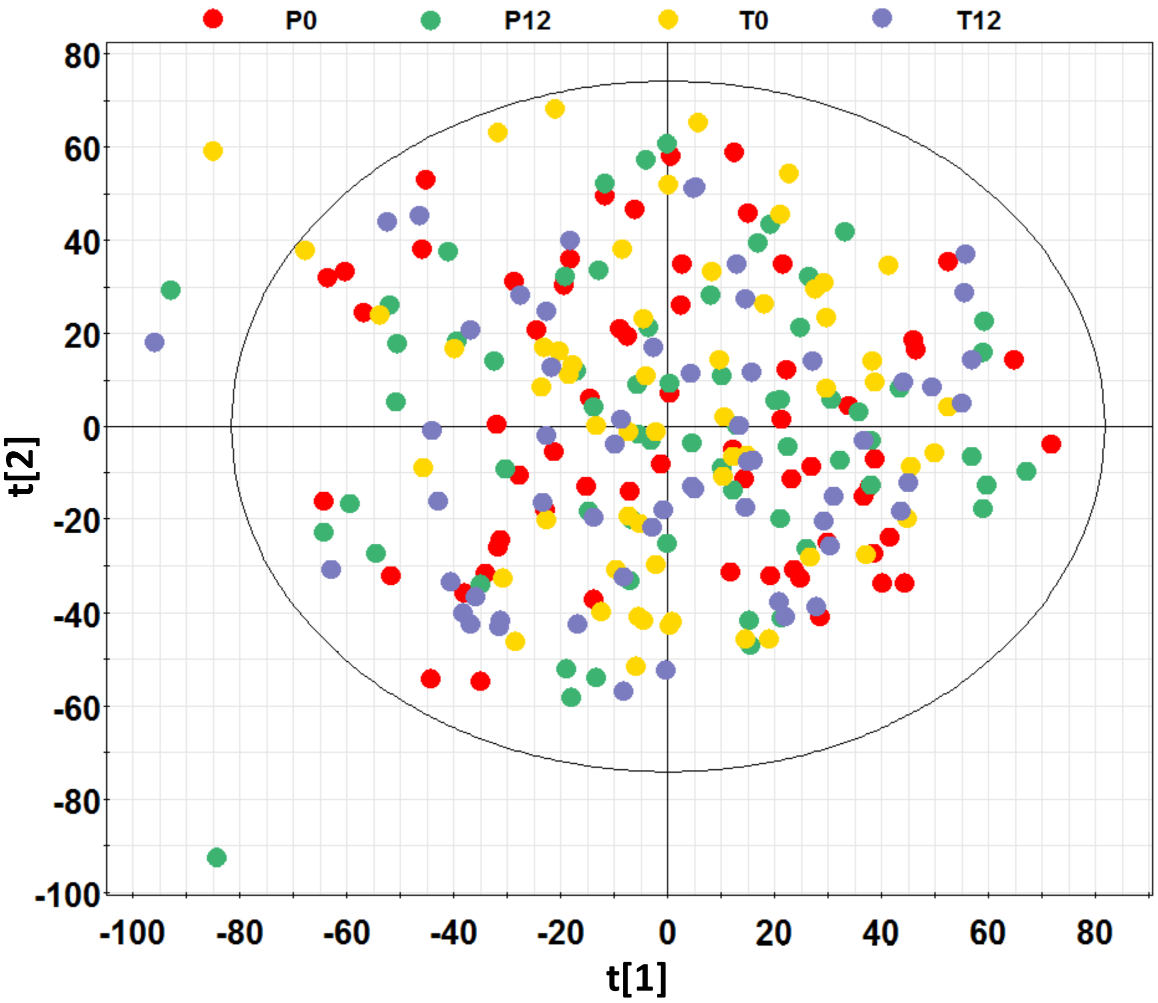

Figure S2. Representative chromatogram of GTP-derived bacterial metabolites in human feces (A) and urine (B).

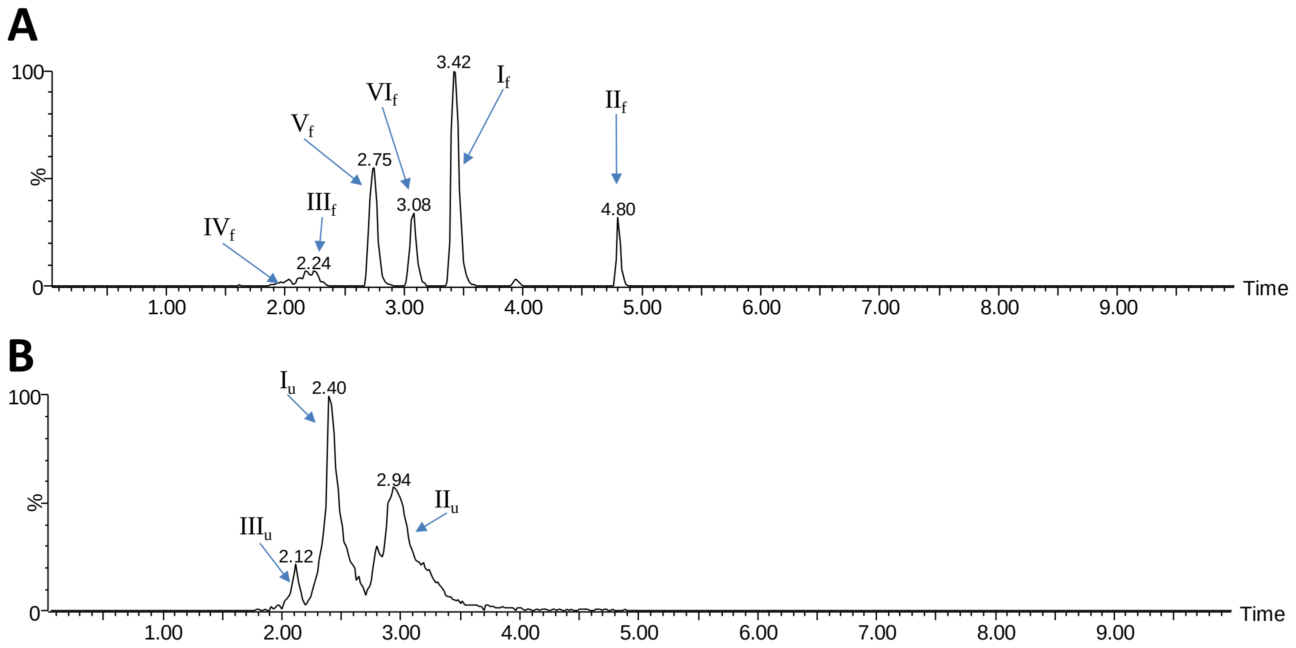

Figure S3. The scores plot from a PCA model on 4 groups of human urine samples, including P0 (before placebo treatment), P12 (after 12-month placebo treatment), T0 (before GTP treatment), and T12 (after 12-month GTP treatment). The t[1] and t[2] are the projection values of each sample in the first and second principal components of the model, respectively.

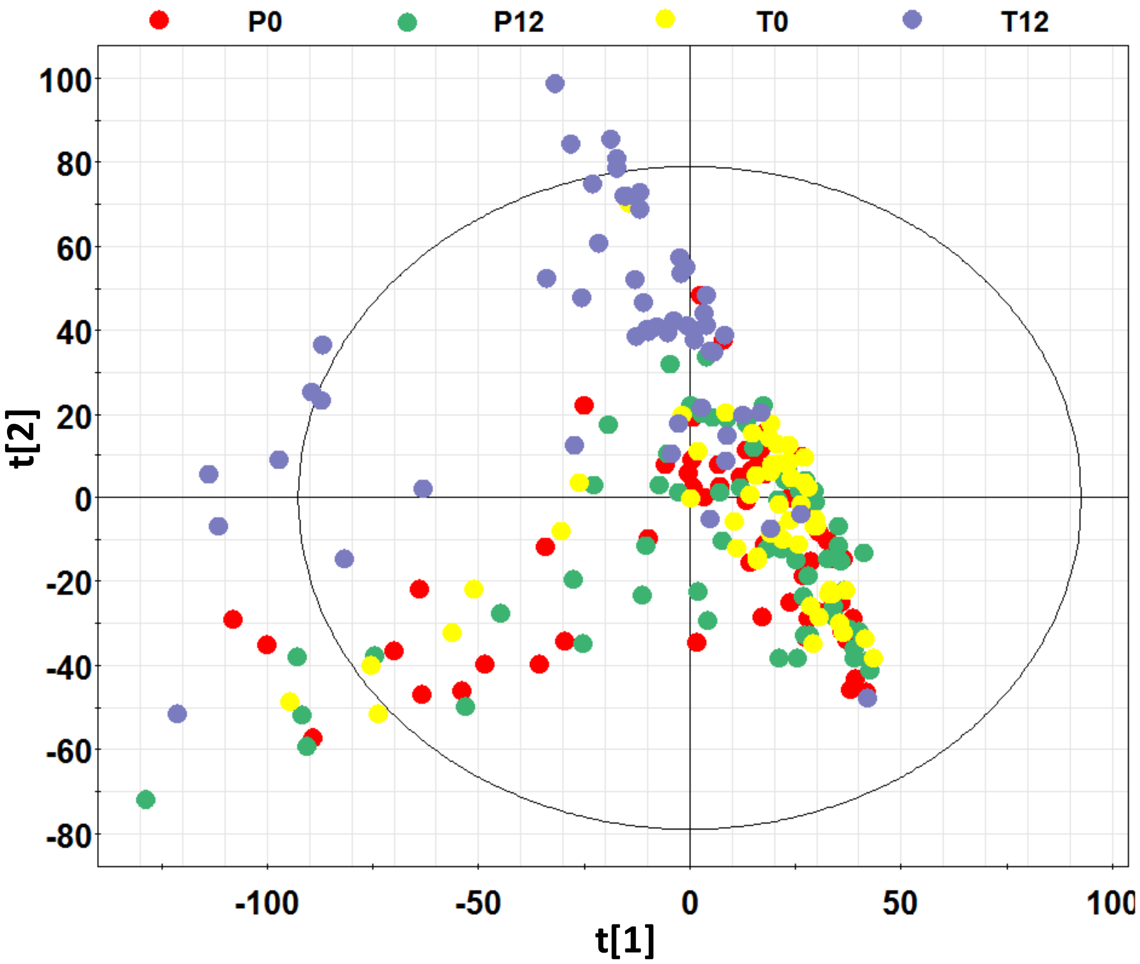

Supplement: Supplementary file 1 [file metabolites-09-00096-s001.pdf]
